# Atherogenic index of plasma as predictors for metabolic syndrome, hypertension and diabetes mellitus in Taiwan citizens: a 9-year longitudinal study

**DOI:** 10.1038/s41598-021-89307-z

**Published:** 2021-05-10

**Authors:** Yen-Wei Li, Tung-Wei Kao, Pi-Kai Chang, Wei-Liang Chen, Li-Wei Wu

**Affiliations:** 1 Department of Psychiatry, Tri‐Service General Hospital and School of Medicine, National Defense Medical Center, Taipei, Taiwan, ROC; 2 Division of Family Medicine, Department of Family and Community Medicine, Tri-Service General Hospital and School of Medicine, National Defense Medical Center, 2F, No. 325, Sec. 2, Cheng-Gong Rd., Neihu district, Taipei City, 114 Taiwan, ROC; 3 Division of Geriatric Medicine, Department of Family and Community Medicine, Tri-Service General Hospital and School of Medicine, National Defense Medical Center, Taipei, Taiwan, ROC; 4 Division of Colon and Rectal Surgery, Department of Surgery, Tri-Service General Hospital and School of Medicine, National Defense Medical Center, Taipei, Taiwan, ROC; 5 Graduate Institute of Medical Sciences, National Defense Medical Center, Taipei, Taiwan, ROC

**Keywords:** Predictive markers, Type 2 diabetes, Hypertension, Metabolic syndrome, Dyslipidaemias

## Abstract

Deeply involved with dyslipidemia, cardiovascular disease has becoming the leading cause of mortality since the early twentieth century in the modern world. Whose correlation with metabolic syndrome (MetS), hypertension and type 2 diabetes mellitus (T2DM) has been well established. We conducted a 9-year longitudinal study to identify the association between easily measured lipid parameters, future MetS, hypertension and T2DM by gender and age distribution. Divided into three groups by age (young age: < 40, middle age: ≥ 40 and < 65 and old age: ≥ 65), 7670 participants, receiving standard medical inspection at Tri-Service General Hospital (TSGH) in Taiwan, had been enrolled in this study. Atherogenic index of plasma (AIP) was a logarithmically transformed ratio of triglyceride (TG)/high-density lipoprotein cholesterol (HDL-C). Through multivariate regression analyses, the hazard ratio (HR) of AIP for MetS, hypertension and T2DM were illustrated. AIP revealed significant association with all the aforementioned diseases through the entire three models for both genders. Additionally, AIP revealed significant correlation which remained still after fully adjustment in MetS, hypertension, and T2DM groups for subjects aged 40–64-year-old. Nevertheless, for participants aged above 65-year-old, AIP only demonstrated significant association in MetS group. Our results explore the promising value of AIP to determine the high-risk subjects, especially meddle-aged ones, having MetS, hypertension, and T2DM in the present and the future.

## Introduction

Deeply involved with dyslipidemia, cardiovascular disease (CVD) has becoming the leading cause of mortality since the early twentieth century in the modern world^1^. Multiple risk factors contributing to CVD such as diabetes, obesity, hypertension, and dyslipidemia has been well established^2^. Among these, plasma lipid profile has been taken regarded as the major risk factor as well as predictor for CVD in the recent years^3^. Dyslipidemia, described as elevation in low-density lipoprotein cholesterol (LDL-C), total cholesterol (TC), and triglyceride (TG) and decreases in high-density lipoprotein cholesterol (HDL-C) has been proved to result in atherosclerosis by a growing number of researches^4^. Among them, LDL-C was thought of as the primary therapeutic target in the past. Nevertheless, about 50% remnant cardiovascular risk remained after lowering LDL-C to the suggested levels. Which encouraged researchers to reach out for new CVD predictors^5^. Our literature review in this area illustrated the significance of numerous lipid ratios or ‘‘atherogenic indexes’’ in the optimization of the predictive power of the lipid profile. For example, TC/HDL-C, LDL-C/HDL-C, non-HDL-C (TC minus HDL-C), non-HDL-C/HDL-C (atherogenic index, AI), and TC ∗ TG ∗ LDL/HDL-C (lipoprotein combine index, LCI) are considered to serve as better predictors for CVD^6^.

Recent studies have indicated that Atherogenic Index of Plasma (AIP), calculated according to the formula, log(TG/HDL-C)^3^, not only reflects the true relationship between protective and atherogenic lipoprotein but also stands out as strong predictor of atherosclerosis and coronary heart disease^7^. However, previous studies revealed discordant results. Hartopo et al.^8^ demonstrated the association between major adverse cardiovascular events and the AIP value (AIP ≥ 0.24 as high AIP group; AIP < 0.24 as low AIP group) in patients suffering from acute myocardial infarction during intensive hospitalization in a prospective cohort study. Their results demonstrated the role of a low instead of high AIP value as an independent predictor for all-cause mortality for their participants. A cross-sectional study in Cameroonian postmenopausal women reported that AIP failed to hold the potential of being an independent risk factor of CVD by Nansseu et al.^9^.

Therefore, aimed to evaluate the correlation between AIP and hypertension, T2DM, and MetS in a Taiwan population, we conducted this longitudinal study to not only identify the promising value of AIP of predicting these diseases of civilization by age distribution but also implement a population strategy for prevention in the future decades.

## Methods and materials

### Study population

For this 9-year longitudinal study between 2007 and 2015, a total of 44,563 participants receiving routine health checkups at the health promotion center of Tri-Service General Hospital (TSGH) in Taiwan had been included for investigation.

**Figure 1:**
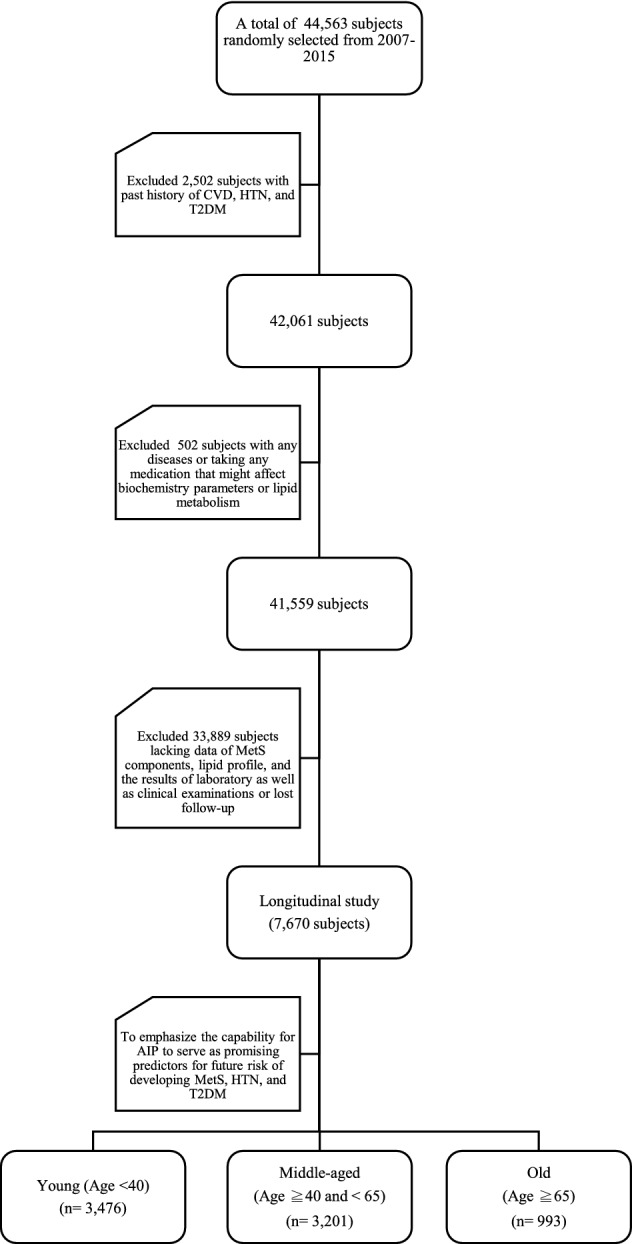
Description of the study design. *CVD* cardiovascular disease, *HTN* hypertension, *MetS* metabolic syndrome, *T2DM* type 2 diabetes mellitus, *AIP* atherogenic index of plasma.

As shown in Fig. 1, to eliminate the influence from possible confounding factors, we have determined exclusion criteria as the followings: subjects with past medical history of CVD, hypertension, T2DM (n = 2502); subjects with any diseases or taking antidiabetic agents, antihypertensive medications, and lipid-lowering drugs that might affect biochemistry parameters or lipid metabolism **(**n = 502**)**. In addition, subjects lacking data of MetS components, lipid profile, and the results of laboratory as well as clinical examinations or lost follow-up **(**n = 33,889) had been excluded.

Consequently, only 7670 participants aged 20–97 years were enrolled in this part of the study. For its well documented changes for prevalence of MetS, hypertension and T2DM in different age groups, we further classified our subjects according to age distribution as young (< 40) (n = 3476), middle-aged (≥ 40 and < 65) (n = 3201), and old (≥ 65) (n = 993). The major goal of this part was to emphasize the capability for AIP to serve as promising predictors for future risk of developing the three aforementioned diseases.

### Anthropometric measurements and general data

Through a questionnaire, our trained nursing staff collected past medical history, family history as well as current medications from each of our participants, respectively. While the complete physical examinations were performed by physicians. Waist circumference (WC) was measured in the standing position at the level of the umbilicus to the nearest 1 cm using a constant tension tape. Body mass index (BMI) was calculated as the subject’s body weight in kilograms divided by the square of the subject’s height in meters (kg/m^2^). Both systolic blood pressure (SBP) and diastolic blood pressure (DBP) were measured in sitting position through standard mercury sphygmomanometers after a 5-min rest period. For each occasion, blood pressure was measured for 2 times and the time interval between successive measurements was 30–60 s to acquire an average of 2 readings^10^.

### Laboratory measurements

To obtain serum levels of TC, TG, HDL-C, LDL-C as well as fasting glucose, peripheral blood of each participant was commenced after an overnight fast about 12 h.

Collected in EDTA containing tubes, blood samples of all participants were acquired through venipuncture procedure at the same room. Fasting blood sugar was measured in duplicate by a glucose oxidase method (YSI 203 glucose analyzer, Scientific Division, Yellow Springs Instruments, Yellow Springs, OH). Through an enzymatic cholesterol assay following dextran sulfate precipitation, concentration of serum LDL-C and HDL-C had been analyzed. In addition, through dry, multilayer analytical slide method in the Fuji Dri-Chem 3000 analyzer (Fuji Photo Film, Minato-Ku, Tokyo, Japan), we’ve obtained concentrations of TG and TC.

### Statistical analysis

All data analysis was performed by IBM SPSS Statistics (SPSS Released 2009. PASW Statistics for Windows, Version 18.0. SPSS). Descriptive statistics were used to analyze the studied parameters and characterize the study subjects. Data were presented as the mean ± SD for quantitative parameters while numbers and percentages for qualitative data. Correlation analysis was carried out by Spearman test and Student’s t test for between-group comparisons. The HRs for the different gender and age groups during the follow-up period had been performed by univariate and multivariate Cox regression models. Statistical significance was suggested by two-tailed p values lower than 0.05.

### Definition

Metabolic syndrome was diagnosed on the basis of International Diabetes Federation Global Consensus Definition^11^ as central obesity (based on race- and gender-specific WC cutoffs; ≥ 90 cm in men and ≥ 80 cm in women for South Asians) coupled with whichever two of the subsequent four criteria as shown in Supplementary Table S1.

Hypertension was defined as the following: (1) SBP ≥ 140 mmHg and/or (2) DBP ≥ 90 mmHg and/or (3) antihypertensive medication usage. Participants with T2DM were diagnosed with the American Diabetes Association criteria: (1) fasting plasma glucose was ≥ 7.0 mmol/L (or 2-h postprandial glucose 0.11.1 mmol/L) and/or (2) whether there was current usage of antidiabetics^12^.

### Ethics approval

Authorized by the Institutional Review Board at TSGH (#2_106_05_144) based on the revised Helsinki Declaration, the data were collected and used only for research purposes. The entire methods were performed conforming to relevant regulations and guidelines. Information on medical records such as past medical history, family history, lifestyle, alcoholic intake, and cigarette smoking were obtained during interviews. An informed consent had been signed by every participant before study participation.

## Results

### Characteristics and Atherogenic Index of Plasma of the participants with or without metabolic syndrome

Baseline characteristics and biochemical variables of our study subjects were summarized in Table 1. Elevated BMI, WC, SBP, DBP, body fat percentage, fasting glucose, TG, TC, LDL-C, BUN, CREA, UA as well as decreased HDL-C were noticed for subjects with MetS. Significant association between AIP and MetS was also demonstrated.

**Table 1:** Demographic data of participants with or without metabolic syndrome.

	Metabolic syndrome (n = 1168)	Control (n = 6502)	p value
**Continuous variables**			
Patient profile, mean (SD)			
Age (years), mean (SD)	50.94 (15.30)	43.84 (15.77)	< 0.001**
BMI (kg/m^2^), mean (SD)	27.78 (4.39)	22.99 (3.36)	< 0.001**
WC (cm), mean (SD)	90.67 (9.32)	77.36 (9.93)	< 0.001**
SBP (mmHg), mean (SD)	127.27 (18.49)	112.63 (20.92)	< 0.001**
DBP (mmHg), mean (SD)	80.94 (12.16)	71.37 (10.97)	< 0.001**
Body fat percentage (%), mean (SD)	30.88 (6.28)	27.07 (6.86)	< 0.001**
Serological data, mean (SD)			
FPG (mg/dL), mean (SD)	114.16 (37.93)	91.57 (16.13)	< 0.001**
HDL-C (mg/dL), mean (SD)	44.44 (10.73)	59.43 (15.54)	< 0.001**
TG(mg/dL), mean (SD)	184.23 (136.25)	97.41 (70.35)	< 0.001**
TC(mg/dL), mean (SD)	193.14 (34.78)	184.31 (33.88)	< 0.001**
LDL-C (mg/dL), mean (SD)	124.41 (32.23)	115.01 (30.92)	< 0.001**
BUN(mg/dL), mean (SD)	14.22 (4.25)	13.19 (4.01)	< 0.001**
CREA(mg/dL), mean (SD)	0.88 (0.21)	0.82 (0.26)	< 0.001**
UA(mg/dL), mean (SD)	6.46 (3.12)	5.40 (1.41)	< 0.001**
Total Ca (mg/dL), mean (SD)	9.20 (0.40)	9.20 (0.39)	0.681
P (mg/dL), mean (SD)	3.97 (1.19)	3.90 (1.73)	0.382
r-GT (U/L), mean (SD)	45.19 (63.38)	26.59 (28.76)	< 0.001**
Alb (g/dL), mean (SD)	4.62 (0.30)	4.64 (0.32)	0.253
A/G ratio, mean (SD)	1.56 (0.27)	1.61 (0.25)	< 0.001**
Direct bilirubin (mg/dL), mean (SD)	0.17 (0.08)	0.18 (0.09)	0.199
CRP(mg/dL), mean (SD)	0.27 (0.29)	0.22 (0.57)	0.230
TSH (uU/mL), mean (SD)	2.41 (2.70)	2.20 (1.90)	0.056
FT4 (ng/dL), mean (SD)	1.29 (0.28)	1.45 (0.44)	0.157
AIP, mean (SD)	0.66 (0.26)	0.21 (0.27)	< 0.001**
**Categorical variables**			
Male, N (%)	985 (64.6)	4251 (48.3)	< 0.001**
CV history, N (%)	320 (28.7)	1533 (24.8)	0.005**

*BMI* body mass index, *WC* waist circumference, *SBP* systolic blood pressure, *DBP* diastolic blood pressure, *FPG* fasting plasma glucose, *HDL-C* high-density lipoprotein cholesterol, *TG* triglyceride, *TC* total cholesterol, *LDL-C* low-density lipoprotein cholesterol, *BUN* blood urea nitrogen, *CREA* creatinine, *UA* uric acid, *Ca* calcium, *P* phosphorus, *r-GT* r-glutamyl transferase, *Alb* albumin, *A/G ratio* albumin/globulin ratio, *CRP* C-Reactive Protein, *TSH* thyroid-stimulating hormone, *FT4* free T4, *AIP* atherogenic index of plasma, *CV* history, family history of cardiovascular disease (three-point major adverse cardiovascular events (3P-MACE) defined as a composite of nonfatal stroke, nonfatal myocardial infarction, and cardiovascular death).

*p < 0.05, **p < 0.01.

### Association between the risk of future metabolic syndrome, hypertension, and type 2 diabetes mellitus as well as Atherogenic Index of Plasma by sex and age

To investigate the role of AIP in prediction of MetS, hypertension and type 2 DM, multivariate Cox regression was performed to evaluate the HRs for future development of MetS, hypertension, and T2DM by gender and age distribution (< 40, 40–64, ≥ 65), respectively. As shown in Table 2, AIP revealed significant association with all the aforementioned diseases through the entire three models for both genders. Except failing to do so in hypertension group with adjustment of Model 3 for female participants.

**Table 2:** Multivariate hazard ratios of future metabolic syndrome, hypertension and type 2 diabetes mellitus by AIP according to sex.

Variables	Model 1		Model 2		Model 3	
	Exp(β) (95% CI)	p value	Exp(β) (95% CI)	p value	Exp(β) (95% CI)	p value
**MetS**						
Male (n = 2146)	13.443 (10.782, 16.762)	< 0.001**	12.166 (9.687, 15.280)	< 0.001**	8.531 (6.614, 11.004)	< 0.001**
Female (n = 2248)	69.146 (45.213, 105.748)	< 0.001**	40.909 (25.379, 65.942)	< 0.001**	38.475 (23.512, 62.961)	< 0.001**
**HTN**						
Male (n = 2146)	2.367 (1.771, 3.164)	< 0.001**	1.966 (1.463, 2.641)	< 0.001**	1.373 (1.033, 1.826)	0.029*
Female (n = 2248)	5.343 (3.502, 8.154)	< 0.001**	1.877 (1.153, 3.055)	0.011*	1.642 (0.997, 2.703)	0.051
**Type 2 DM**						
Male (n = 2205)	5.179 (3.181, 8.432)	< 0.001**	4.972 (3.106, 7.958)	< 0.001**	2.239 (1.373, 3.652)	0.001**
Female (n = 2281)	39.114 (17.623, 86.813)	< 0.001**	12.731 (4.956, 32.703)	< 0.001**	9.472 (3.296, 27.221)	< 0.001**

Adjusted covariates:

Model 1 = Unadjusted.

Model 2 = Model 1 + age, sex and body mass index (BMI).

Model 3 = Model 2 + CREA (creatinine), SBP (systolic blood pressure), FPG (fasting plasma glucose).

*AIP* atherogenic index of plasma, *MetS* metabolic syndrome, *HTN* hypertension, *DM* diabetes mellitus.

*p < 0.05, **p < 0.01.

For subjects aged < 40-year-old (Table 3), significant association between MetS, hypertension and T2DM as well as AIP was noticed originally but failed in T2DM group with Model 3 adjustment.

**Table 3:** Multivariate hazard ratios of future metabolic syndrome, hypertension and type 2 diabetes mellitus by AIP according to age.

Variables	Model 1		Model 2		Model 3	
	Exp(β) (95% CI)	p value	Exp(β) (95% CI)	p value	Exp(β) (95% CI)	p value
**MetS**						
Age = 1–39 (n = 2207)	58.225 (39.459, 85.915)	< 0.001**	47.683 (29.432, 77.253)	< 0.001**	46.010 (27.430, 77.173)	< 0.001**
Age = 40–64 (n = 1998)	15.301 (12.267, 19.084)	< 0.001**	12.698 (9.930, 16.238)	< 0.001**	8.886 (6.780, 11.647)	< 0.001**
Age > 65 (n = 189)	11.700 (4.639, 29.510)	< 0.001**	12.199 (4.712, 31.584)	< 0.001**	14.461 (4.521, 46.255)	< 0.001**
**HTN**						
Age = 1–39 (n = 2207)	9.184 (5.974, 14.121)	< 0.001**	3.311 (1.972, 5.558)	< 0.001**	2.529 (1.460, 4.379)	0.001**
Age = 40–64 (n = 1998)	2.887 (2.182, 3.821)	< 0.001**	1.904 (1.388, 2.610)	< 0.001**	1.467 (1.088, 1.980)	0.012*
Age > 65 (n = 189)	0.798 (0.317, 2.011)	0.633	0.732 (0.287, 1.865)	0.513	0.935 (0.323, 2.704)	0.901
**Type 2 DM**						
Age = 1–39 (n = 2079)	26.759 (8.678, 82.517)	< 0.001**	19.839 (4.954, 79.440)	< 0.001**	2.360 (0.398, 14.004)	0.345
Age = 40–64 (n = 2055)	7.410 (4.842, 11.339)	< 0.001**	5.936 (3.794, 9.288)	< 0.001**	2.464 (1.534, 3.957)	< 0.001**
Age > 65 (n = 197)	5.006 (1.160, 21.598)	0.031*	5.540 (1.206, 25.461)	0.028*	6.005 (0.900, 40.060)	0.064

Adjusted covariates:

Model 1 = Unadjusted.

Model 2 = Model 1 + age, sex and body mass index (BMI).

Model 3 = Model 2 + CREA (creatinine), SBP (systolic blood pressure), FPG (fasting plasma glucose).

*AIP* atherogenic index of plasma, *MetS* metabolic syndrome, *HTN* hypertension, *DM* diabetes mellitus.

*p < 0.05, **p < 0.01.

AIP revealed significant correlation which remained still after fully adjustment in MetS, hypertension, and T2DM groups for subjects aged 40–64-year-old (Table 3). However, for participants aged above 65-year-old, AIP only demonstrated significant association in MetS group (Table 3).

## Discussion

The aim of the present study was to investigate the possible contribution of AIP to future hypertension, T2DM, and MetS in Taiwan citizens between 2007 and 2015. Potential age-dependent relationship between AIP and risk of developing the three diseases mentioned above was also demonstrated.

In the present study, the significant association between AIP and MetS was consistent with previous studies with a smaller cohort of participants^13,14^. A sex-modulated association of AIP with MetS, hypertension, and T2DM presented in previous study demonstrated a gender difference concerning incident hypertension, with a considerably weaker association in female than in male^15^. Which resembles our observations in female in the current research. The above discovery indicates that there are other mediators which take a more central part in female. It has been proposed that vascular adhesion molecules, dysfunctional HDL-C particles or its apoproteins dysfunctional adiponectin, complement C3, etc., might be influential as well^16–18^. It is well established that the prevalence of MetS, hypertension and T2DM all rise from young to old ages^19–22^. Our results demonstrated that AIP revealed significant correlation with all the aforementioned diseases for the entire three models for subjects aged 40–64-year-old. However, AIP failed to maintain the above relationship with the other age subgroups. As far as the author could review, there were few reports focusing on the relationship between AIP and risk of future MetS, hypertension, and T2DM by age distribution. There is also paucity of literature on why AIP failed to maintain the above relationship with the other age subgroups. Nevertheless, a 7.8-year follow-up study of 2676 middle-aged adults showed that AIP is a reliable biomarker for predicting diabetes and hypertension^15^. Essiarab et al. demonstrated the significant association between AIP and metabolic syndrome (MetS) in muddle-aged participants^14^, which was consistent with our finding.

The biological mechanisms for higher AIP causing increasing risk for MetS might be explained through dyslipidemia. As a well-known risk factor for CVD, dyslipidemia also plays an important role in MetS for both TG and HDL-C serving as relevant diagnostic criteria of it. From the physiopathology point of view, remaining particles, rich in TG, had been demonstrated their contribution to both the formation and progression of atheromatous plaque^23^. Besides their function of the reverse transport of cholesterol, HDL-C particles show a broad spectrum of promising biological activities such as being antiatherosclerotics^24^. The combination of hypertriglyceridemia and decreased levels of HDL-C (which means elevated AIP) not only holds strong relationship to increasing risk of CVD but also serves as the more frequently found lipid profile among overweight adolescents^25,26^. Which further emphasized the role of AIP playing in preventive medicine.

Composed of elevated concentrations of plasma TG, decrease in HDL-C and advantage on quality of small, dense low-density lipoprotein (sdLDL), diabetic dyslipidemia had been well discussed in recent years^27^. By means of contesting with glucose for entrance to the cell that result in impairment of glucose oxidation, hypertriglyceridemia may result in insulin resistance. Besides, increasing levels of TG downgrade both the amount and activity of insulin receptors on adipocytes^28^. Decreased concentrations of HDL-C take a negative part in β-cell function through decline the sensitivity and secretion of insulin. On the other hand, elevation in levels of TG and free fatty acids along with declined HDL-C could result from insulin resistance. The above two interact as both cause and effect, which illustrated the dyslipidemia-insulin resistance-hyperinsulinemia “vicious cycle” hypothesis of T2DM^29^. Easier than LDL-C to combine with glycoprotein, deposit on arterial wall, and result in impaired clearance of plasma lipoprotein particles, sdLDL had been proved its crucial role in arteriosclerosis and cardiovascular disease^27^.

With the highest sensitivity for predicting acute coronary events^30^, the predictive value for AIP plays in CVD had been well documented^31,32^. Previous study^33^ demonstrated a practicable merit of utilizing AIP is that its values range from negative to positive with zero closely corresponding to the diameter of LDL-C of 25.5 nm, which was the size determined as a cut-off between LDL-C phenotype A and B in prior research^34^. Dobiásová et al. conjecture that AIP demonstrates an equilibrium between the definite concentration of serum HDL-C and TG which might predetermine the direction of cholesterol transport in intra-vascular pool^35^. Besides, AIP was reported to be the most sensitive marker compared with other three atherogenic indices like Castelli’s risk index-I (TC/HDL-C), Castelli’s risk index-II (LDL-C/HDL-C), and atherogenic coefficient (TC-HDL-C/HDL-C)^36,37^. Furthermore, in circumstances where all atherogenic parameters are normal, AIP might serve as the alternative screening method^38^. Even though in the above study most of participants (77.5%) were in the elevated risk AIP group, a large number of them did not possess any risk factor. Nevertheless, there is paucity of literature on the timing of determining AIP in the context of preventive measures to prevent the development of cardiometabolic diseases. Indeed, it has been suggested that AIP values of − 0.3 to 0.1 are associated with low, 0.1–0.24 with medium and above 0.24 with high risk of CVD^39^. As far as we know, both the present study and previous researches failed to demonstrate whether people with high risk score develop metabolic cardiometabolic comorbidity earlier. Nevertheless, we demonstrated optimal AIP cut-off values for predicting the presence of MetS, hypertension and T2DM through Youden's index in conjunction with receiver operating characteristic (ROC) analyses in different sex and age groups (Supplementary Tables S2, S3). To the best of our knowledge, the main strength of this research is that this is the first longitudinal study in Taiwan to identify the potential of AIP on the prediction of future MetS, hypertension, and T2DM in different age subgroups. Nevertheless, several limitations still existed in our study. First, the participants of our study were not from a general population, but from a health promotion center located in the capital city of Taiwan so they may not be typical of the general population for relatively higher socio-economic status. Second, cytokines (such as IL-6 and TNF-α), apolipoproteins (such as Apo-A1 and Apo-B) and other metabolic parameters were not measured in this study because of budget shortage. Third, we did not have accessible data of cardiovascular event or all-cause mortality, which could influence the interpretation of our results. Fourth, a number of participants were excluded on account of incomplete data or lost follow-up; therefore, selection bias was noted in the present reasearch. At last, the definite timing of developing incident MetS, hypertension, and T2DM was unavailable in the present study, which might provide AIP with stronger predictive value for the development of cardiometabolic diseases. Future studies with other metabolic parameters, circulating levels of cytokines and apolipoproteins, and incidence about cardiovascular events and/or all-cause mortality could provide further assistance and insights into our present discoveries.

In conclusion, our results explore the promising value of AIP to determine the high-risk subjects, especially meddle-aged ones, having MetS, hypertension, and T2DM in the present and the future. Moreover, AIP should be taken as a regular monitoring index of CVD in every day practice, especially for those already having other CVD risks. Higher levels of these easily measured parameters of lipid profile may take important parts in these modern diseases, which special attention should be paid by healthcare providers, from the present into the future.

## Supplementary Information

Supplementary Information 1.

## Abbreviations

AIP: Atherogenic index of plasma
Apo-A1: Apolipoprotein A1
Apo-B: Apolipoprotein B
BMI: Body mass index
BUN: Blood urea nitrogen
CREA: Creatinine
CVD: Cardiovascular disease
DBP: Diastolic blood pressure
EDTA: Ethylenediaminetetraacetic acid
HDL-C: High-density lipoprotein cholesterol
IL-6: Interleukin-6
LDL-C: Low-density lipoprotein cholesterol
MetS: Metabolic syndrome
ROC: Receiver operating characteristic
SBP: Systolic blood pressure
sdLDL: Small, dense low-density lipoprotein
TC: Total cholesterol
T2DM: Type 2 diabetes mellitus
TG: Triacylglycerol
TNF-α: Tumor necrosis factor-alpha
UA: Uric acid
WC: Waist circumference
