# Supplementary material for: Atherogenic index of plasma as predictors for metabolic syndrome, hypertension and diabetes mellitus in Taiwan citizens: a 9-year longitudinal study
Source: Sci Rep. 2021 May 10;11:9900. doi: 10.1038/s41598-021-89307-z (PMC8110777; doi:10.1038/s41598-021-89307-z)
Supplement: Supplementary file 1 — Supplementary Information 1. [file 41598_2021_89307_MOESM1_ESM.docx]

**Atherogenic index of plasma as predictors for metabolic syndrome, hypertension and diabetes mellitus in** **Taiwan citizens: a 9-year longitudinal study**

Yen-Wei Li^1^, Tung-Wei Kao^2,3^, Pi-Kai Chang^4,5^, Wei-Liang Chen^2,3,5^, Li-Wei Wu^2,3,5^

^1^Department of Psychiatry, Tri‐Service General Hospital; and School of Medicine, National Defense Medical Center, Taipei, Taiwan, Republic of China.

^2^Division of Family Medicine, Department of Family and Community Medicine, Tri-Service General Hospital; and School of Medicine, National Defense Medical Center, Taipei, Taiwan, Republic of China.

^3^Division of Geriatric Medicine, Department of Family and Community Medicine, Tri-Service General Hospital; and School of Medicine, National Defense Medical Center, Taipei, Taiwan, Republic of China.

^4^Division of Colon and Rectal Surgery, Department of Surgery, Tri-Service General Hospital; and School of Medicine, National Defense Medical Center, Taipei, Taiwan, Republic of China.

^5^Graduate Institute of Medical Sciences, National Defense Medical Center, Taipei, Taiwan, Republic of China.

**Correspondence to**: Li-Wei Wu, M.D.

^*^Division of Family Medicine, Division of Geriatric Medicine, Department of Family and Community Medicine, Tri-Service General Hospital, National Defense Medical Center, 2F, No. 325, Sec. 2, Cheng-Gong Rd., Neihu district, Taipei city 114, Taiwan (R.O.C.).

Tel: +886-2-87923311 ext. 16567

Fax: +886-2-87927057

E-mail: bigmouth0825@hotmail.com

**Supplementary Table 1. Subsequent criteria of metabolic syndrome.**

| Subject | Definition |
| --- | --- |
| Elevated triglycerides | ≧150 mg/dL (≧1.7 mmol/L) or history of specific therapeutic management for this lipid abnormality. |
| Decreased HDL-C | <40 mg/dL (<1.03 mmol/L) in men and <50 mg/dL (<1.29 mmol/L) in women or history of specific therapeutic management for this lipid aberrance. |
| Elevated blood pressure | SBP ≧130 mm Hg or DBP ≧85 mmHg or on treatment for previously diagnosed hypertension. |
| Elevated fasting glucose | ≧100 mg/dL (≧5.6 mmol/L) or previously diagnosed T2DM. |

Abbreviation:

HDL-C, high-density lipoprotein cholesterol; SBP, systolic blood pressure; DBP, diastolic blood pressure; T2DM, type 2 diabetes mellitus

**Supplementary Table 2. Optimal AIP cut-off values for predicting the presence of metabolic syndrome, hypertension and type 2 diabetes mellitus in different gender group.**

|  | MetS | | HTN | | Type 2 DM | |
| --- | --- | --- | --- | --- | --- | --- |
|  | Male | Female | Male | Female | Male | Female |
| AUC (95%CI) | 0.876 (0.864, 0.887) | 0.911 (0.899, 0.923) | 0.586 (0.567, 0.605) | 0.673 (0.651, 0.695) | 0.678 (0.649, 0.707) | 0.804 (0.773, 0.836) |
| Sensitivity (%) | 85.3 | 87.2 | 52.2 | 72.4 | 69.2 | 75.6 |
| Specificity (%) | 76.1 | 81.7 | 61.6 | 54.6 | 59.7 | 75.0 |
| P-value | <0.001 | <0.001 | <0.001 | <0.001 | <0.001 | <0.001 |
| Cut-off value | 0.488 | 0.332 | 0.439 | 0.139 | 0.429 | 0.322 |

Abbreviation:

AIP, atherogenic index of plasma; MetS, metabolic syndrome; HTN, hypertension; DM, diabetes mellitus

**Supplementary Table 3. Optimal AIP cut-off values for predicting the presence of metabolic syndrome, hypertension and type 2 diabetes mellitus in different age group.**

|  | MetS | | | HTN | | | Type 2 DM | | |
| --- | --- | --- | --- | --- | --- | --- | --- | --- | --- |
|  | Age = 1-39 | Age = 40-64 | Age > 65 | Age = 1-39 | Age = 40-64 | Age > 65 | Age = 1-39 | Age = 40-64 | Age > 65 |
| AUC (95%CI) | 0.925 (0.911, 0.938) | 0.882 (0.868, 0.896) | 0.849 (0.820, 0.878) | 0.720 (0.691, 0.749) | 0.611 (0.588, 0.634) | 0.530 (0.490, 0.569) | 0.831 (0.760, 0.902) | 0.707 (0.672, 0.741) | 0.703 (0.656, 0.751) |
| Sensitivity (%) | 87.0 | 79.7 | 71.9 | 70.0 | 59.3 | 33.7 | 80.5 | 75.2 | 76.1 |
| Specificity (%) | 85.1 | 81.5 | 84.8 | 65.6 | 58.9 | 75.1 | 78.1 | 58.8 | 60.5 |
| P-value | <0.001 | <0.001 | <0.001 | <0.001 | <0.001 | 0.140 | <0.001 | <0.001 | <0.001 |
| Cut-off value | 0.425 | 0.473 | 0.451 | 0.265 | 0.344 | 0.458 | 0.418 | 0.356 | 0.331 |

Abbreviation:

AIP, atherogenic index of plasma; MetS, metabolic syndrome; HTN, hypertension; DM, diabetes mellitus
